# Effect of Synthetic Pregelatinized Starch-Modified C-S-H Particles on the Chemical Structure of C-A-S-H Generated from GGBS

**DOI:** 10.3390/ma16175736

**Published:** 2023-08-22

**Authors:** Weijie Hao, Zheyu Zhu

**Affiliations:** 1 School of Construction Engineering, Zhengzhou Shengda University, Zhengzhou 451191, China; 15713783078@163.com; 2 School of Materials Science and Engineering, Yancheng Institute of Technology, Yancheng 224051, China

**Keywords:** calcium silicate hydrate, pregelatinized starch, modification, slag, GGBS

## Abstract

Finding new agents to enhance the strength of alkali-activated (ground granulated blast furnace slag) GGBS is beneficial for environmental protection. Here, we reveal the effect of pregelatinized starch-modifying calcium silicate hydrate (C-S-H) particles on the nanostructure tailoring of NaOH-activated GGBS hydrates. The results show that, for the synthetic modified C-S-H, the pregelatinized starch absorbs on the surface of C-S-H, which modifies the silicate chains and crystal structure. Adding pregelatinized starch-modified C-S-H particles can tailor the chemical structure of calcium silicaluminate hydrate (C-A-S-H) formed from GGBS hydration by increasing the mean chain length (MCL) and decreasing the Al/Si ratios. When adding C-S-H particles modified by 0.1% pregelatinized starch, the MCL of C-A-S-H is increased by 344.5% and the Al/Si ratio is decreased by 16.0%. The compressive strength of NaOH-activated GGBS samples can be enhanced by adding pregelatinized starch-modified C-S-H particles, while the addition for modified C-S-H does not significantly affect the flexural strength. The high strength of hardened blocks of hydrated GGBS is related to the long MCL silicate chains. These findings provide a potential application of pregelatinized starch-modifying C-S-H particle acting as strength-enhancing agents.

## 1. Introduction

Concrete is the most widely applied building material in the world, which contributes around 5~8% of total CO_2_ emission [1,2,3,4]. GGBS is a by-product of blast furnace smelting of the metallurgical industry. The replacement of cement in building materials by ground granulated blast furnace slag (GGBS) facilitates reducing the environmental impact of CO_2_ emission from cement production [5,6]. Recent studies show that GGBS meets the requirements of the activity [7,8] and is also usually applied as a supplement of Portland cement, which contributes to improving the performance of cement such as strength [9], anti-salt attack [9] and carbonation [10,11,12]. GGBS can be activated by activators (e.g., alkaline hydroxides and salts) and become hardened blocks [13,14,15]. For alkaline hydroxides activators, the limited application of GGBS is related to its high addition of alkali. Theoretically, when decreasing such addition, the low strength of GGBS at early age (e.g., curing for 3 d) occurs. Thus, finding new ways to enhance the strength of GGBS with a low addition of alkali is beneficial for technological upgrading and environmental protection.

From the perspective of the GGBS hardening mechanism, the activator can generate calcium silicate hydrate (C-S-H), which is the source of activated GGBS strength. Up to now, the discovered C-S-H varies with about 40 types in cementitious materials. The nanostructure of classic C-S-H was considered to be the aggregates of tobermorite-like nanocrystals, jennite-like nanocrystals and disordered gels. This nanostructure was easily distorted by numerous factors such as calcium to silicon (Ca/Si) ratio, water to cement (w/t) ratio and impurity ions (e.g., aluminum, magnesium and sodium). In GGBS the alkaline oxides CaO and MgO, and acidic oxides Al_2_O_3_ and SiO_2_ are the main compositions [7,16], which can generate C-A-S-H and N-C-S-H by alkali activated processes [14,17,18,19,20]. The C-A-S-H forms through aluminum-oxide tetrahedra connecting with silicate tetrahedra [21,22]. Compared with the C-S-H in Portland cement, the C-A-S-H and N-C-S-H in GGBS forms by cationic substitution. Theoretically, if the replacement of Al^3+^ for Si^4+^ occurs at bridging positions, a charge imbalance is generated. This charge imbalance is compensated by positive ions such as Na^+^ ions, Mg^2+^ ions and Ca^2+^ ions in gels. The N-C-S-H forms by the sodium ions balancing the charges of silicates chains. The properties of GGBS are directly related to the nature and structure of C-A-S-H gels. Compared to C-S-H chains in Portland cement, the chains in GGBS hydrates are longer, since aluminum-oxide tetrahedra make up silicate chains defects in C-S-H [21]. In cement chemistry, the chemical environment of ^29^Si in silicates depends on the most recent coordination number Q^n^, where n (usually 0–4) is the number of oxygen atoms shared by each tetrahedron with adjacent tetrahedra [23,24,25]. Different activators can cause the various Q^n^ distribution of silicates chains in GGBS hydrates. For example, the waterglass induces the C-A-S-H gels with high Q^2^, Q^3^ or Q^Poly^ contents [26,27], and NaOH solution induces the C-A-S-H gels to exhibit a high Q^2^ unit content [26]. Another activator such as Na_2_CO_3_ salts generate C-A-S-H gels dominated by Q^1^ and Q^2^ units [28]. In addition, the carbonate salts also act as strength-enhancing agents, which improves compressive strength.

Recent references show that different activators can cause the nanostructure of GGBS hydrates to be dominated by certain Q^n^ chains. However, research on C-A-S-H chain tailoring in GGBS hydrates is still lacking. Since the properties of materials depend on their structure, the method proposed here tailors the micro–nano structure through introducing synthetic C-S-H into activated GGBS. Considering the source of activated GGBS is relative to C-A-S-H generation, the addition of synthetic C-S-H in the hydration of GGBS has the potential to enhance the early strength of alkali-activated GGBS.

Through the use of bottom-up nanotechnology, the nanostructure of C-S-H has been modified and the healing of defects has been critical to the improvement in performance. The synthesis method of “polymer-inserting C-S-H” [29] and “polymer binding C-S-H” [23] are usually adopted to obtain different nanostructures of C-S-H. For example, polyvinyl alcohol (PVA), polyethylene glycol (PEG), polyaniline, polyacrylic acid and hydroxyl-terminated polydimethylsiloxane (PDMS) have been used to prepare polymer/C-S-H particles at the nanoscale [30,31,32,33,34,35,36,37,38]. This provides the referable technology to achieve targeted C-S-H with a specific nanostructure. Pregelatinized starch is a biodegradable polymer which is produced cheaply. The nanostructure of the pregelatinized starch has some active chemical bonds from breaking the intermolecular bonds and connecting the hydrogen bonding sites (hydroxyl and oxygen). Zhu [39] demonstrated that those active chemical bonds in pregelatinized corn-starch have the potential to modify the C-S-H nanostructure.

Inspired by this, this paper synthesized three classic pregelatinized corn-starch modifying C-S-H particles with different Q^n^ distribution firstly. And then, the chemical structure and morphology of the three classic C-S-H particles were confirmed by XRD, FTIR, NMR and SEM. After this, three classic C-S-H particles were introduced to the NaOH-activated GGBS materials. The mechanism of microstructure tailoring of GGBS hydrates were revealed. FTIR and NMR were used to obtained the distribution changes of the Q^n^ distribution in silicate chains. To verify the effect of synthetic C-S-H, the mechanical properties were also evaluated. This research provides a cheap and environmentally friendly method to enhance activated GGBS materials.

## 2. Materials and Methods

### 2.1. Synthesis of Pregelatinized Corn-Starch/C-S-H Samples

The C-S-H samples were synthesized by mixing aqueous hydrated sodium silicate solutions (Na_2_SiO_4_·9H_2_O; Sigma-Aldrich (St. Louis, MO, USA), ≥98%; 1.81 g powder dissolved in 50 g water), pregelatinized starch solution (dissolved in 100 g water) and hydrated calcium nitrate (Ca(NO_3_)_2_·4H_2_O; Sigma-Aldrich, 99%; 2.18 g powder dissolved in 50 g water). An amount of 2 g NaOH solid powders was added to adjust the pH to 12.2. The Ca/Si molar ratio of the synthetic pregelatinized starch-modifying C-S-H samples was 1.0. In the mixing process, solution A was prepared by first mixing the pregelatinized starch solution with the Na_2_SiO_4_·9H_2_O solution. The Ca(NO_3_)_2_·4H_2_O solution was then added to solution A with stirring and a white precipitate formed immediately. The sample was hardened at 60 °C for 24 h with stirring. Before characterization, the samples were washed twice with 1000 mL deionized water and then dried at 40 °C. The addition of pregelatinized maize starch was 0.0%, 0.1% and 0.3% by mass of solids (the sum of Na_2_SiO_4_·9H_2_O and Ca(NO_3_)_2_·4H_2_O powders), which was designated C-S-H, STACSH01 and STACSH03, respectively. The binding property of pregelatinized maize starch is relative to the hydrogen bonding sites (hydroxyl hydrogen and oxygen). The main chemical composition of GGBS is shown in Table 1.

### 2.2. Preparation of GGBS Mortar and Pastes

NaOH has been widely used as activator for obtaining hardened GGBS blocks [15]. To clarify the effects of pregelatinized starch-modified C-S-H particles on GGBS hydrates, the basic activator NaOH is adopted as activator in this research. The GGBS mortar was prepared by mixing pregelatinized starch-modified C-S-H particles, 0.45 kg slag powders, 1.0 kg standard and water. The ratio of water to slag was 0.55. The concentration of NaOH was adjusted to 2 M. The fresh and homogeneous mortars were immediately cast into 40 mm × 40 mm × 160 mm molds and then vibrated for 60 s to remove remaining air bubbles by using a vibration table. Subsequently, the mortars were sealed with a plastic foil and cured for 24 h at 25 °C. The hardened mortars were demolded and cured under standard conditions (20 ± 2 °C, 95 ± 3%RH) until 3 d, 14 d and 28 d. To research the silicate chains of C-A-S-H formed by GGBS, the pastes were prepared by mixing pregelatinized starch-modified C-S-H particles, slag powders and water, and the remaining processes were consistent with the mortar preparation. The sample without adding pregelatinized starch-modified C-S-H particles was labeled G0. And the samples which added pure C-S-H, STACSH01 and STACSH03 were labeled as PCSH0, PCSH01 and PCSH03, respectively.

### 2.3. Testing Procedures

Strengths were tested on the basis of Chinese standard GB/T 17671-1999. The compressive and flexural strengths of hardened GGBS mortars were measured by using a WHY-300 Machine with an axial loading of 2 kN/s and 50 N/s, respectively. Six replicate samples were applied to obtain the average value and error bar of compressive strengths. And three replicate samples were used to obtain the flexural strength average value and error bar. The maximum value and the minimum value were used in the error bar.

### 2.4. Characterization

XRD data were obtained using a Rigaku-D/max 2550VB3 + X-ray diffractometer with graphite-monochromated Cu Kα radiation (λ = 1.541 Å) generated at 40 kV and 100 mA. Scanning was performed with 2θ in the range of 5° to 70° and a scanning speed of 5°/min. The infrared absorption spectra of the samples were analyzed using FTIR (Bruker Tensor 27, Borken, Germany) using the KBr pellet method. ^29^Si MAS NMR experiments were performed on an Agilent 600 DD2 spectrometer at a resonance frequency of 119.23 MHz. The ^29^Si NMR cross-polarization (CP) spectra were recorded using a 4 mm probe at a spin rate of 15 kHz at room temperature. Si CPMAS experiments were performed with a delay time of 3 s and a contact time of 1 ms using 5000 scans. Deconvolution was used to assign resonances to individual species using a combination of Gaussian and Lorentzian functions. The micromorphology of the samples was observed using SEM (FEI QUANTA 200FEG-ESEM, Morgan Hill, CA, USA). The porosity and pore structure of GGBS mortars after 3 days of curing were characterized by the MIP method using AutoPore Iv 9510 (McPrittyk, Norcross, GA, USA). The mean chain length (MCL) and the Al/Si ratio were calculated using Equations (1) and (2), respectively [40,41].

(1) $$\mathrm{MC}L=\frac{2\left[Q^{1}+{\;Q}^{2\left(0\mathrm{Al}\right)}+\frac{3}{2}Q^{2\left(1\mathrm{Al}\right)}+{\;Q}^{3\left(0\mathrm{Al}\right)}+{\;Q}^{3\left(1\mathrm{Al}\right)}\right]}{Q^{1}}$$

(2) $$\mathrm{Al}/\mathrm{Si}=\frac{\frac{1}{2}Q^{2\left(1\mathrm{Al}\right)}}{Q^{1}+Q^{2\left(0\mathrm{Al}\right)}+{\;Q}^{2\left(1\mathrm{Al}\right)}+{\;Q}^{3\left(0\mathrm{Al}\right)}+{\;Q}^{3\left(1\mathrm{Al}\right)}}$$

## 3. Results and Discussion

### 3.1. Structure of Pregelatinized Starch/C-S-H Particles

#### 3.1.1. XRD

**Figure 1:**
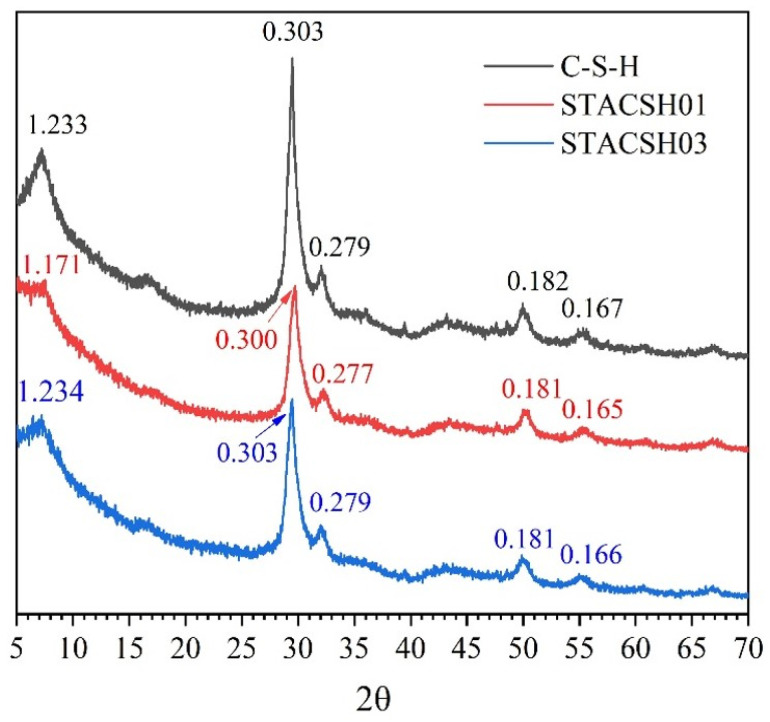
XRD patterns of C-S-H and pregelatinized starch-modified C-S-H particles.

Figure 1 shows the XRD analysis of pregelatinized starch-modified C-S-H particles. In Figure 1, the d-spacing of synthesized C-S-H is 1.233, 0.303, 0.279, 0.182 and 0.167 nm, which is close to tobermorite (PDF# 83-1520). It is clearly observed that the XRD peaks of pregelatinized starch-modified C-S-H particles were changed to broaden at d-spacing of 1.171 nm for STACSH01 and 1.234 nm for STACSH03. This indicates that the nanostructure of C-S-H was distorted by pregelatinized starch resulting in a poor layered structure. Theoretically, there are two explanations for this phenomenon. One is that pregelatinized starch absorbs on the surface of C-S-H units. Jennings described the C-S-H as the aggregates of 3~5 nm crystals-like units. If pregelatinized starch absorbs on the surface of nanoscale C-S-H units, it may distort the surface atom arrangement and then the inner atom arrangement is changed. The other explanation is that pregelatinized starch inserts into the layers of tobermorite-like units which happens with low probability. Tobermorite is a layered crystal where the layer spacing is 9 Å, 11 Å or 14 Å. From this view, the layer spacing of synthesized C-S-H is about 12.3 Å in this study. If pregelatinized starch inserts into the layers of C-S-H units, the d-spacing which represents (0 0 2) planes (~6°) should became larger. However, the d-spacing is 11.7 Å and 12.3 Å (peaks at ~6°) in pregelatinized starch/C-S-H particles, which indicates a low probability of insertion. In addition, the d-spacing at 0.300 nm for STACSH01 samples demonstrates the designed effect of pregelatinized starch on the nanostructure C-S-H particles. Thus, there are three synthesized C-S-H with different nanostructure obtained by addition of pregelatinized starch. In order to explore the effect of pregelatinized starch on the chemical bond interaction and silicate chains of C-S-H, the FTIR and NMR measurements were adopted.

#### 3.1.2. FTIR

**Figure 2:**
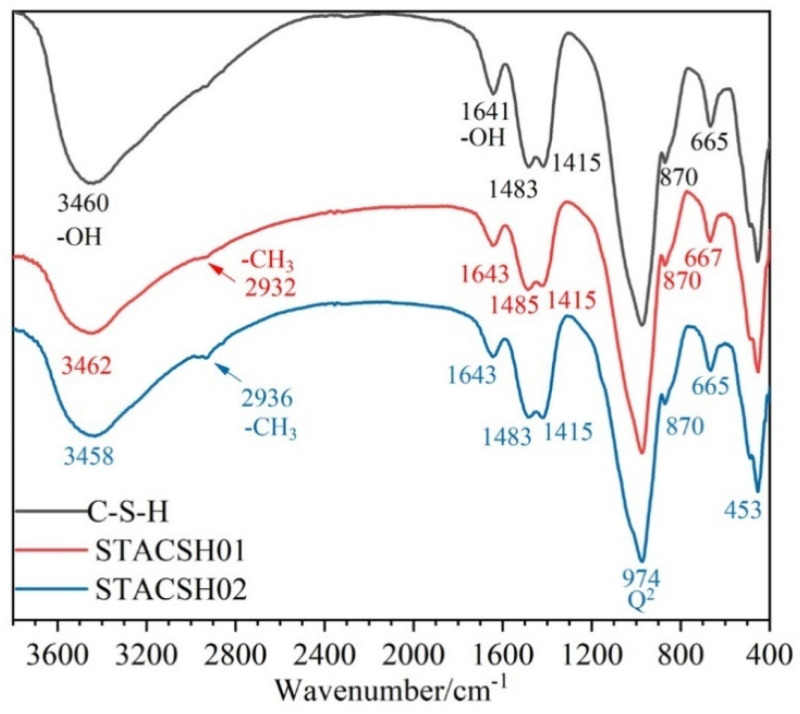
FTIR spectra of C-S-H and pregelatinized starch-modified C-S-H particles.

Figure 2 shows the FTIR analysis of pregelatinized starch-modifying C-S-H particles (C-S-H, STACSH01, and STACSH03 samples). In Figure 2, the C-S-H, STACSH01 and STACSH03 samples have bands at around 665 cm^−1^ due to Si-O-Si bending vibrations. The band at 974 cm^−1^ is assigned to the Si-O stretching vibrations of Q^2^ tetrahedra. The bands at around 1640 cm^−1^ and 3460 cm^−1^ belong to stretching vibrations of -OH groups in H_2_O or hydroxyls. The group of bands near 447 cm^−1^ belong to the deformation of silicate tetrahedra. In the STACSH01 and STACSH03 samples, the 2932 cm^−1^ and 2936 cm^−1^ bands correspond to methyl groups (-CH_3_) [23,39,42]. It can be clearly observed that, with increasing pregelatinized starch concentration, the absorption of -CH_3_ bonds increases gradually. There are no new chemical bonds of Si-O-R (R represent other groups) existing in the STACSH01 and STACSH03 samples, which demonstrates that pregelatinized starch may absorb on the surface of nanoscale C-S-H units. Other evidence for this absorption is the shifting of -OH bands at 1643 cm^−1^, 3462 cm^−1^ and 3458 cm^−1^ which is due to hydrogen bonding. Krautwurst [43] pointed out that C-S-H is formed by two steps that follow the non-classical crystallization theory; that is, C-S-H precursor forms firstly and then evolves into crystal units. Zhu [23] described a mixture of PDMS and C-S-H unit nanostructure, where PDMS deposited in the void space left by aggregates of nanocrystalline C-S-H. XRD analysis shows that pregelatinized starch changes the nanostructure of C-S-H units. FTIR shows this process does not form the new chemical bonds of Si-O-R but a physical action. In this research, pregelatinized starch may also distort the aggregates of C-S-H units during the formation of C-S-H.

#### 3.1.3. NMR

**Figure 3:**
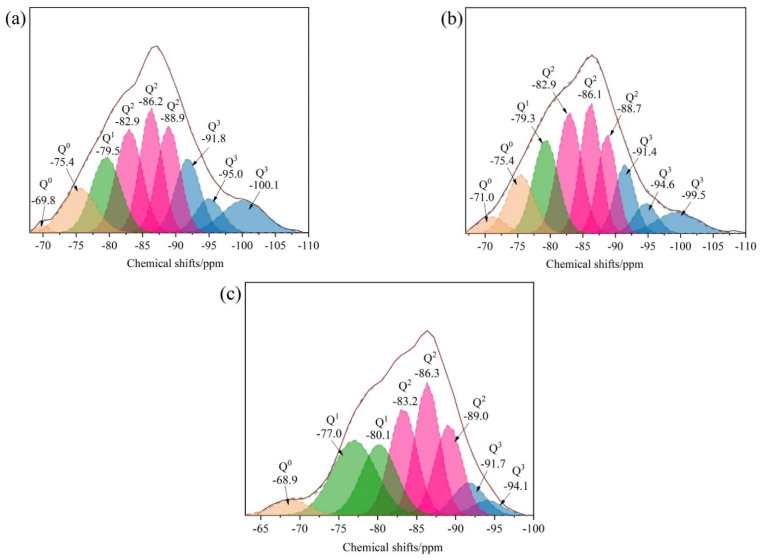
^29^Si NMR spectra of C-S-H and pregelatinized starch/C-S-H particles. (**a**) C-S-H, (**b**) SATCSH01 samples and (**c**) SATCSH03 samples.

Figure 3a shows the ^29^Si NMR spectra of C-S-H. Figure 3b shows the ^29^Si NMR spectra of STACSH01. Figure 3c shows the ^29^Si NMR spectra of STACSH03. Peak fitting is performed before and after the pregelatinized starch modification to obtain the Q^n^ distribution of silicate chains. The chemical shifts of −78.0 to −82.5 ppm belongs to Q^1^, −84.0 to −89.0 ppm belongs to Q^2^ and −89.0 to −104 ppm belongs to Q^3^ [23,24,25,33]. Two distinct chemical environments of Q^2^, the major peak at around −84.5 ppm and overlapped peak at around −89.5 ppm, were found in all samples. Table 2 shows the chemical shifts and contents of Q species in the C-S-H and STACSH samples. For C-S-H samples, the content of Q^2^ is 45.3% + 50.4% = 95.7% and the content of Q^3^ is 4.3%. For STACSH01 samples, the content of Q^2^ is 39.6% + 47.9% = 87.5%, and the content of Q^1^ and Q^3^ is 3.1% and 9.5%, respectively. For the STACSH03 samples, the content of Q^2^ is 39.2% + 50.7% = 89.9%, and the content of Q^1^ and Q^3^ is 5.1% and 4.4%, respectively. Compared with C-S-H, the addition of 0.1% pregelatinized starch increases the content of Q^3^ silicate chains by 120.9% in STACSH01 samples and, when the addition of pregelatinized starch is 0.3% (STACSH03 samples), the content of the Q^3^ tetrahedron decreases to be similar to C-S-H. In addition, the content of the Q^1^ tetrahedron increases with the addition of pregelatinized starch. This indicates that the pregelatinized starch content affects and rearranges the nanostructure of C-S-H silicate chains. Theoretically, the C-S-H with a high content of Q^3^ tetrahedra has a better performance than that with a low content of Q^3^ tetrahedra. Yang Zhou [33] found that Q^3^ enhanced the Young’s modulus according to the nanoindentation measurement by destroying the layered structure. The XRD results show that the layered pregelatinized starch/C-S-H particle structure was distorted. However, in our research the STACSH03 samples have the same content of Q^3^ as the C-S-H sample, which indicates that layered structure changes are not due to Q^3^ formation but the absorption of pregelatinized starch. J.J. Beaudoin [24] demonstrated that polymers can simulate Q^3^ signals by forming polymer–Q^2^ groups. If the pregelatinized starch bonds with C-S-H via Si-O-R, the Q^3^ content increases with decreasing of Q^2^. This explanation does not match the NMR analysis of the STACSH03 sample nanostructure. Thus, the speculation about pregelatinized starch absorbing on the surface of C-S-H and then modifying the nanostructure of silicate chains matches the results of XRD, FTIR and NMR.

#### 3.1.4. SEM

**Figure 4:**
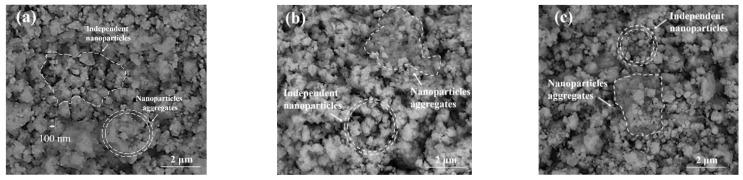
SEM micrographs of C-S-H and pregelatinized starch/C-S-H particles. (**a**) C-S-H, (**b**) SATCSH01 samples and (**c**) SATCSH03 samples.

Figure 4a shows the microtopography of C-S-H particles. Figure 4b shows microscopic topographical views of STACSH01 samples. Figure 4c shows microscopic topographical views of STACSH03 samples. It was clearly observed that C-S-H is made up of aggregates of particles with the sizes ranging from about 100 nm to around 1000 nm. Scholars [23,33] have pointed out that polymers such as PVA, polyaniline and PDMS change the microscopic morphology of C-S-H to a cauliflower-like structure. In Figure 4b,c, the STACSH01 and STACSH03 samples are also made up of aggregates of particles with similar sizes to the C-S-H samples. There are two different regions existing in the microtopography of pregelatinized starch-modified C-S-H particles. One is a region consisting of around 300~500 nm independent nanoparticles and the other is a compact nanoparticle aggregate region. The observed phases existing between the adjacent particles are used for differentiating those two regions. And those two regions can also be observed in Figure 4a. This indicates pregelatinized starch does not affect the microtopography of C-S-H, which is due to physical action between pregelatinized starch and C-S-H units. Pregelatinized starch is considered to dissolve in water by destroying the double helix structure of starch. According to the XRD, FTIR and NMR results, the unfolded molecular chain absorbs on the surface of C-S-H particles of various sizes. The unchanged microtopography before and after modification indicates that pregelatinized starch participates in the stacking of C-S-H but does not form continuous polymer films. Pregelatinized starch may play a role of adhesion among the C-S-H particles at nanoscale.

### 3.2. Effect of Pregelatinized Starch/C-S-H Particles on the C-A-S-H Nanostructure Formed from the Alkali Activated GGBS

#### 3.2.1. Chemical Bond Analysis

**Figure 5:**
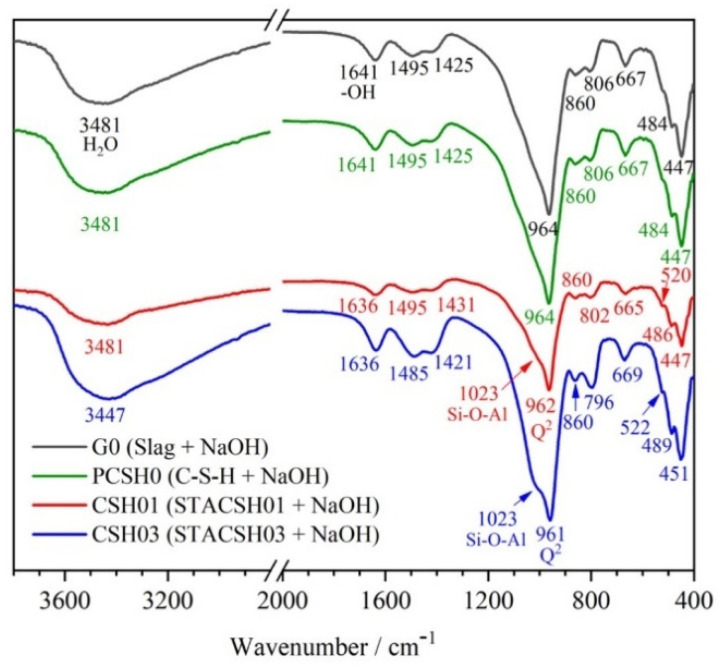
FTIR spectra of GGBS modified by pregelatinized starch-modified C-S-H cured for 3 d.

Figure 5 shows the FTIR spectrum of GGBS modified by pregelatinized starch/C-S-H additives which were cured for 3 d. In Figure 2, the bands at about 667 cm^−1^ and 964 cm^−1^ belong to Q^2^ tetrahedra. The bands at around 1640 cm^−1^ and 3480 cm^−1^ belongs to -OH groups in H_2_O or hydroxyls. It is noteworthy that bands at about 520 cm^−1^, 800 cm^−1^ and 1023 cm^−1^, which represent Al-O-Al, Si-O-Al and Si-O-Al bonds in C-(A)-S-H, respectively [38], appeared in samples with pregelatinized starch/C-S-H additives samples CSH01 and CSH03. In actuality, those Al-O-Al and Si-O-Al bonds also exist in the G0 and PCSH0 sample in our research. The failed detection of 520 cm^−1^ and 1023 cm^−1^ signals was due to their low content, and the supplementary information was obtained by NMR below. The FTIR results demonstrated silicate chain tailoring of GGBS by addition of pregelatinized starch-modified C-S-H particles. Since the performance is crucial relative to the material’s nanostructure, the C-A-S-H nanostructure change dominates the strength of GGBS at the molecular level. In order to obtained the detail of silicate chains, a NMR spectrometry analysis was conducted below.

#### 3.2.2. Silicate Chain Nanostructure

**Figure 6:**
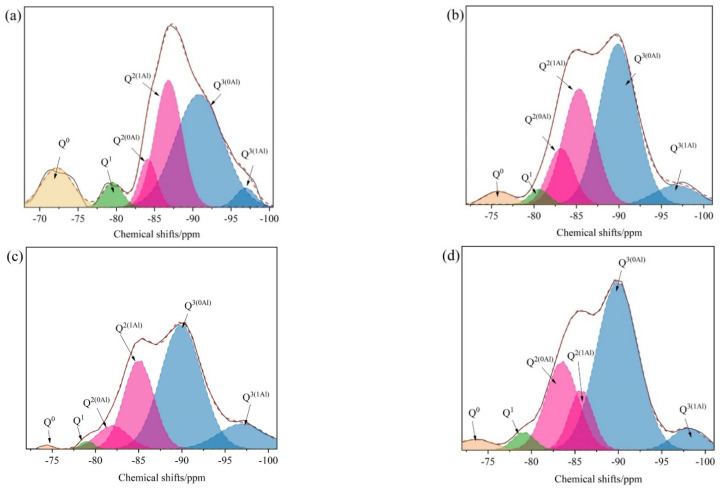
NMR spectrometry analysis of GGBS modified by pregelatinized starch-modified C-S-H cured for 3 d. (**a**) G0; (**b**) PCSH0; (**c**) PCSH01; (**d**) PCSH03.

Figure 6 and Table 3 show the NMR analysis of GGBS modified by pregelatinized starch-modified C-S-H additives. For all the activators, the NMR spectra can be deconvoluted into six peaks. These chemical shift peaks were attributed to: Q^0^ (−68.0 to −76.0 ppm), Q^1^ (−78.0 to −82.0 ppm), Q^2(0Al)^ (around −83.5 ppm), Q^2(1Al)^ (around −85.5 ppm), Q^3(0Al)^ (around −90.0 ppm) and Q^3(1Al)^ (around −97.5 ppm) [40,44]. In Figure 6a, when only activated by NaOH, the Q^0^, Q^1^, Q^2^ and Q^3^ content is 10.4%, 4.1%, 7.1% + 29.1% = 36.1% and 46.0% + 3.3% = 49.3%, respectively. In Figure 6b–d, when adding synthetic C-S-H particles, the corresponding Q^0^ contents are 2.9%, 0.5% and 3.1%, the corresponding Q^1^ contents are 2.3%, 1.0% and 3.0%, the corresponding Q^2^ contents are 40.2%, 34.4% and 33.4%, and the corresponding Q^3^ contents are 54.4%, 64.1% and 60.4%. The synthetic C-S-H particles affect the Q^n^ distribution of C-A-S-H generated in the NaOH-activated GGBS systems. The addition of PCSH0, CSH01 and CSH03 decreases the content of Q^0^ species, which belongs to the anhydrous GGBS. This indicates synthetic C-S-H particles enhance the NaOH activation process. Another finding is that, compared to the G0 sample, the content of Q^2(1Al)^ also decreases in the PCSH0, CSH01 and CSH03 samples, which demonstrates that the replacement of Al^3+^ for Si^4+^ can be tailored by adding pregelatinized starch-modified C-S-H particles. To determinate the detailed nanostructure of silicate chains of hydrates, the MCL value and the Al/Si ratio were evaluated. In Table 4, the addition of synthetic C-S-H particles affects the MCL and Al/Si ratio. Compared with G0 samples, the MCL values of PSCH0, PCSH01 and PCSH03 are increased by 90.7%, 344.5% and 34.3%, respectively. And the Al/Si ratio are decreased by 7.5%, 16.0% and 64.19%, respectively. Theoretically, the long MCL value can lead to better mechanical properties of C-S-H. The early strength enhancement of GGBS mortars was verified by adding synthetic C-S-H particles and the results are discussed as below.

According to the recent finding [41,45], C-S-H is considered to follow a non-classical crystalline process, where the amorphous nano-sized particles are formed first followed by the formation of crystals. This crystalline pathway is heteronucleation [41,45]; that is, the crystalline phase may form at the active site of the amorphous precursor [45]. This non-classical crystalline theory can be referenced here to explain the tailoring effects on C-A-S-H growth by pregelatinized starch-modifying C-S-H. When the silico-oxygen tetrahedron in GGBS is stripped by NaOH, the silico-oxygen tetrahedron (Q^0^) is dissolved in an alkaline solution. At that time, the early precursors formed by the polymerization of silicate chains are the main source of strength of GGBS. The application of modified C-S-H to GGBS resulted in a significant reduction in the Q^0^ content of CSH01 and CSH03 during the experiment, probably due to the heteronucleation process causing Q^0^ chains to accumulate on the different active sites of modified C-S-H with a different structure. We speculate that the pregelatinized starch-modified C-S-H particles induce the formation of the C-A-S-H precursor with a specific structure and the subsequent reaction is controlled by a sufficient number of precursors, which determines the final chemical structure of C-A-S-H. This process appears to affect the C-A-S-H structure by influencing the Al/Si ratio, which can be verified by the NMR analysis. In summary, the addition of modified C-S-H particles affects the precipitation process of the polymerized silico-oxygen tetrahedra. And the precursor will first nucleate and grow at the active site of the externally modified C-S-H. Since the microstructural change can theoretically enhance the macroscopic mechanical properties, optimizing the mechanical properties can be achieved by increasing the MCL value, which is verified below. The MCL of C-A-S-H increased due to the structural adaptation of these active sites, which represents a promising new approach to GGBS activation.

### 3.3. Verification

**Figure 7:**
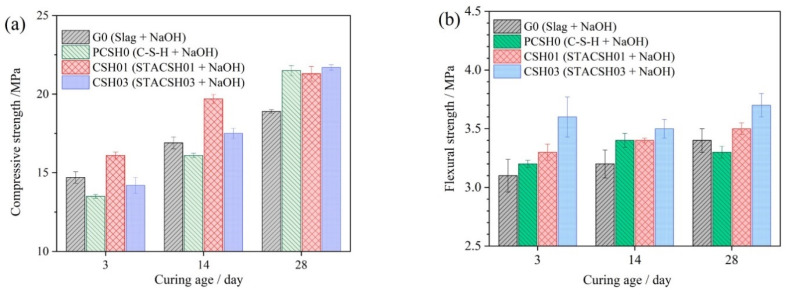
Mechanical strength of GGBS modified by pregelatinized starch/C-S-H additives. (**a**) Compressive strength and (**b**) flexural strength.

Figure 7 gives the compressive and flexural strength development of GGBS by addition of pregelatinized starch/C-S-H particles. In Figure 7, the early strength of GGBS samples was cured for 3 d. In order to verify that the early strength was not attenuated, the samples cured for 14 d and 28 d were also prepared. It is observed in Figure 7a that there is evident enhancement of early compressive strength by addition of STACSH01 particles which occupy 0.88% of GGBS powders by mass. Compared with the G0 sample, the enhanced compressive strength of the CSH01 samples is 9.52% for 3 d curing, 16.6% for 14 d curing and 12.7% for 28 d curing. Compared with the G0 samples, the addition of C-S-H and STACSH03 particles decreases the compressive strength for 3 d and 14 d curing. This demonstrates the early enhancing action is related to the optimized special structure of STACSH01 particles. It is noteworthy that the addition of C-S-H and STACSH03 enhanced the 28d compressive strength by 14.8%, which indicates potential enhancement of long age curing. In Figure 7b, the flexural strength is around the same as all samples cured for 3 d, 7 d and 28 d. The addition of modified C-S-H does not affect the flexural strength. In summary, the results demonstrate the pregelatinized starch-modifying C-S-H has a special effect on the early strength of GGBS hydrates. Since the strength of this system originates from C-A-S-H generation, the explanation for early performance of GGBS is related to the different Q^n^ distribution of silicate chains. A similar phenomenon can be seen in nanosilica-enhanced GGBS hardened blocks, where nanosilica increases the 28 d compressive strength by 8.3–19.22% [46]. The nanocrystal seeds also play an enhancing role in compressive strength. In this research, the size of pregelatinized starch-modified C-S-H particles is at a micron scale; their actions may be different from the nanosilica seeds. That is, the nucleation active sites discussed above in modified C-S-H particles may play a significant role in early strength enhancement.

## 4. Conclusions

To enhance the understanding of basic mechanisms in tailoring nanostructures of activated GGBS system with lower NaOH usage, pregelatinized starch-modified C-S-H particles were synthesized, firstly, and then introduced into the GGBS hydration process. The nanostructures of both pregelatinized starch-modifying C-S-H and C-A-S-H generated from GGBS hydration were revealed. And the potential application in strength enhancement was also evaluated.

Three synthesized C-S-H with different nanostructures can be obtained by the addition of pregelatinized starch. The pregelatinized starch absorbs on the surface of C-S-H and changes the silicate chains and crystal structure. The chemical structure of generated C-A-S-H from GGBS hydration can be tailored by pregelatinized starch-modified C-S-H particles. When adding C-S-H particles modified by 0.1% pregelatinized starch, the MCL is increased by 344.5% and the Al/Si ratios decreased by 16.0%. Compared to the pure NaOH-activated GGBS sample, the compressive strength of samples with added C-S-H particles modified by 0.1% pregelatinized starch was improved by 9.5% for 3 d, 16.6% for 14 d and 12.7% for 28 d. And the long MCL silicate chain may cause high strength in hardened blocks of hydrated GGBS. The potential application of pregelatinized starch-modifying C-S-H particles as strength-enhancing agents can be confirmed.

## Figures and Tables

**Table 1:** The main chemical composition of GGBS.

CaO	SiO_2_	Al_2_O_3_	MgO	MgO	Fe_2_O_3_	TiO_2_	K_2_O	Na_2_O
50.8	24.5	15.0	4.8	1.7	0.5	0.9	0.3	0.2

**Table 2:** The chemical shifts and contents of Q species in C-S-H and SATCSH samples.

Sample	Q^1^ (%)		Q^2b^ (%)		Q^2p^ (%)		Q^3^ (%)	
	Chemical Shift /ppm	Content /%	Chemical Shift /ppm	Content /%	Chemical Shift /ppm	Content /%	Chemical Shift /ppm	Content /%
C-S-H	-	-	−84.5	45.3	−89.5	50.4	−96.3	4.3
STACSH01	−81.4	3.1	−85.1	39.6	−89.9,	47.9	−96.8	9.5
STACSH03	−81.0	5.7	−85.0	39.2	−89.9	50.7	−97.4	4.4

**Table 3:** Chemical shifts and contents of Q species in hydrated GGBS samples.

No.	Q^0^		Q^1^		Q^2(0Al)^		Q^2(1Al)^		Q^3(0Al)^		Q^3(1Al)^	
	Shift /ppm	Content /%	Shift /ppm	Content /%	Shift /ppm	Content /%	Shift /ppm	Content /%	Shift /ppm	Content /%	Shift /ppm	Content /%
G0	−72.3	10.4	−79.5	4.1	−84.1	7.1	−86.8	29.1	−90.8	46.0	−96.9	3.3
PCSH0	−75.8	2.9	−80.6	2.3	−83.2	11.1	−85.3	29.1	−89.8	47.1	−96.7	7.3
CSH01	−74.3	0.5	−79.0	1.0	−82.6	7.3	−85.0	27.0	−89.9	51.9	−96.9	12.2
CSH03	−73.5	3.1	−79.1	3.0	−83.5	22.2	−85.7	11.2	−89.9	54.8	−98.0	5.6

**Table 4:** Mean chain length (MCL) and Al/Si ratio of hydrated GGBS samples.

Samples	MCL	Al/Si Ratio
G0	50.8	0.162
PCSH0	96.9	0.150
CSH01	225.8	0.136
CSH03	68.2	0.058

## Data Availability

Not applicable.

## References

[1] Monteiro P.J.M., Miller S.A., Horvath A. (2017). Towards sustainable concrete. Nat. Mater..

[2] Lee H.C., Siew K.W., Gimbun J., Cheng C.K. (2014). Synthesis and characterisation of cement clinker-supported nickel catalyst for glycerol dry reforming. Chem. Eng. J..

[3] Xu S., Chen Z., Zhang B., Yu J., Zhang F., Evans D.G. (2009). Facile preparation of pure CaAl-layered double hydroxides and their application as a hardening accelerator in concrete. Chem. Eng. J..

[4] Zou F., Zhang M., Hu C., Wang F., Hu S. (2021). Novel C-A-S-H/PCE nanocomposites: Design, characterization and the effect on cement hydration. Chem. Eng. J..

[5] Kanchanason V., Plank J. (2019). Effect of calcium silicate hydrate—Polycarboxylate ether (C-S-H–PCE) nanocomposite as accelerating admixture on early strength enhancement of slag and calcined clay blended cements. Cem. Concr. Res..

[6] Schneider M., Romer M., Tschudin M., Bolio H. (2011). Sustainable cement production—Present and future. Cem. Concr. Res..

[7] Zhang L., Jia Y., Shu H., Zhang L., Lu X., Bai F., Zhao Q., Tian D. (2021). The effect of basicity of modified ground granulated blast furnace slag on its denitration performance. J. Clean. Prod..

[8] McCaslin E.R., White C.E. (2021). A parametric study of accelerated carbonation in alkali-activated slag. Cem. Concr. Res..

[9] Guo Z., Wang Y., Hou P., Shao Y., Zuo X., Li Q., Xie N., Cheng X. (2019). Comparison study on the sulfate attack resistivity of cement-based materials modified with nanoSiO_2_ and conventional SCMs: Mechanical strength and volume stability. Constr. Build. Mater..

[10] Jiang W., Li X., Lv Y., Jiang D., Liu Z., He C. (2020). Mechanical and hydration properties of low clinker cement containing high volume superfine blast furnace slag and nano silica. Constr. Build. Mater..

[11] Zhang Z., Wang Q., Chen H., Zhou Y. (2017). Influence of the initial moist curing time on the sulfate attack resistance of concretes with different binders. Constr. Build. Mater..

[12] Güneyisi E., Özturan T., Gesoǧlu M. (2007). Effect of initial curing on chloride ingress and corrosion resistance characteristics of concretes made with plain and blended cements. Build. Environ..

[13] Ye H., Chen Z., Huang L. (2019). Mechanism of sulfate attack on alkali-activated slag: The role of activator composition. Cem. Concr. Res..

[14] Nuno C., João C., Tiago M., Ángel P., Ana F. (2019). Alkali activated composites—An innovative concept using iron and steel slag as both precursor and aggregate. Cem. Concr. Compos..

[15] Sajedi F., Razak H.A. (2010). The effect of chemical activators on early strength of ordinary Portland cement-slag mortars. Constr. Build. Mater..

[16] Li Y.-H., Chang F.-M., Huang B., Song Y.-P., Zhao H.-Y., Wang K.-J. (2020). Activated carbon preparation from pyrolysis char of sewage sludge and its adsorption performance for organic compounds in sewage. Fuel.

[17] Dung N.T., Hooper T.J.N., Unluer C. (2021). Improving the carbonation resistance of Na2CO3-activated slag mixes via the use of reactive MgO and nucleation seeding. Cem. Concr. Compos..

[18] Bernal S.A., Provis J.L., Myers R.J., San Nicolas R., van Deventer J.S.J. (2014). Role of carbonates in the chemical evolution of sodium carbonate-activated slag binders. Mater. Struct..

[19] Garcia-Lodeiro I., Palomo A., Fernández-Jiménez A., Macphee D.E. (2011). Compatibility studies between N-A-S-H and C-A-S-H gels. Study in the ternary diagram Na_2_O–CaO–Al_2_O_3_–SiO_2_–H_2_O. Cem. Concr. Res..

[20] Engelhardt G. (1989). Multinuclear solid-state NMR in silicate and zeolite chemistry. TrAC Trends Anal. Chem..

[21] Puertas F., Palacios M., Manzano H., Dolado J.S., Rico A., Rodríguez J. (2011). A model for the C-A-S-H gel formed in alkali-activated slag cements. J. Eur. Ceram. Soc..

[22] García-Lodeiro I., Cherfa N., Zibouche F., Fernández-Jimenez A., Palomo A. (2014). The role of aluminium in alkali-activated bentonites. Mater. Struct..

[23] Zhu Z., Wang Z., Zhou Y., Wei Y., She A. (2021). Synthesis and structure of calcium silicate hydrate (C-S-H) modified by hydroxyl-terminated polydimethylsiloxane (PDMS). Constr. Build. Mater..

[24] Beaudoin J.J., Raki L., Alizadeh R. (2009). A 29Si MAS NMR study of modified C–S–H nanostructures. Cem. Concr. Compos..

[25] Schneider J., Cincotto M.A., Panepucci H. (2001). 29Si and 27Al high-resolution NMR characterization of calcium silicate hydrate phases in activated blast-furnace slag pastes. Cem. Concr. Res..

[26] Fernández-Jiménez A., Puertas F., Sobrados I., Sanz J. (2003). Structure of Calcium Silicate Hydrates Formed in Alkaline-Activated Slag: Influence of the Type of Alkaline Activator. J. Am. Ceram. Soc..

[27] Brough A.R., Atkinson A. (2002). Sodium silicate-based, alkali-activated slag mortars: Part I. Strength, hydration and microstructure. Cem. Concr. Res..

[28] Dung N.T., Hooper T.J.N., Unluer C. (2019). Accelerating the reaction kinetics and improving the performance of Na_2_CO_3_-activated GGBS mixes. Cem. Concr. Res..

[29] Matsuyama H., Young J.F. (1999). Synthesis of calcium silicate hydrate/polymer complexes: Part I. Anionic and nonionic polymers. J. Mater. Res..

[30] Lü L., Ping B., He Y., He L., Wu X., Hu S. (2014). Effect of polymer on morphology and structure of calcium silicate hydrate prepared via precipitation method. J. Wuhan Univ. Technol.-Mater. Sci. Ed..

[31] Pelisser F., Gleize P.J.P., Mikowski A. (2014). Structure and micro-nanomechanical characterization of synthetic calcium–silicate–hydrate with Poly(Vinyl Alcohol). Cem. Concr. Compos..

[32] Beaudoin J.J., Dramé H., Raki L., Alizadeh R. (2009). Formation and properties of C-S-H–PEG nano-structures. Mater. Struct..

[33] Zhou Y., She W., Hou D., Yin B., Chang H., Jiang J., Li J. (2018). Modification of incorporation and in-situ polymerization of aniline on the nano-structure and meso-structure of calcium silicate hydrates. Constr. Build. Mater..

[34] Mojumdar S.C., Raki L. (2006). Preparation, thermal, spectral and microscopic studies of calcium silicate hydrate–poly(acrylicacid) nanocomposite materials. J. Therm. Anal. Calorim..

[35] Khoshnazar R., Beaudoin J., Raki L., Alizadeh A. (2014). Volume Stability of Calcium-Silicate-Hydrate/Polyaniline Nanocomposites in Aqueous Salt Solutions. ACI Mater. J..

[36] Li H., Xue Z., Liang G., Wu K., Dong B., Wang W. (2021). Effect of C-S-Hs-PCE and sodium sulfate on the hydration kinetics and mechanical properties of cement paste. Constr. Build. Mater..

[37] Xu C., Li H., Yang X., Dong B., Wang W. (2021). Action of the combined presence of C-S-Hs-PCE and triethanolamine on the performances of cement paste/mortar. Constr. Build. Mater..

[38] Kapeluszna E., Kotwica Ł., Różycka A., Gołek Ł. (2017). Incorporation of Al in C-A-S-H gels with various Ca/Si and Al/Si ratio: Microstructural and structural characteristics with DTA/TG, XRD, FTIR and TEM analysis. Constr. Build. Mater..

[39] Zhu Z., Wang Z., Zhou Y., Chen Y., Zhou L., She A. (2021). Evaluation of the nanostructure of calcium silicate hydrate based on atomic force microscopy-infrared spectroscopy experiments. Nanotechnol. Rev..

[40] Zhang G., Yang H., Ju C., Yang Y. (2020). Novel selection of environment-friendly cementitious materials for winter construction: Alkali-activated slag/Portland cement. J. Clean. Prod..

[41] Zhu X., Qian C., He B., Chen Q., Jiang Z. (2020). Experimental study on the stability of C-S-H nanostructures with varying bulk CaO/SiO2 ratios under cryogenic attack. Cem. Concr. Res..

[42] Higl J., Hinder D., Rathgeber C., Ramming B., Lindén M. (2021). Detailed in situ ATR-FTIR spectroscopy study of the early stages of C-S-H formation during hydration of monoclinic C3S. Cem. Concr. Res..

[43] Krautwurst N., Nicoleau L., Dietzsch M., Lieberwirth I., Labbez C., Fernandez-Martinez A., Van Driessche A.E.S., Barton B., Leukel S., Tremel W. (2018). Two-Step Nucleation Process of Calcium Silicate Hydrate, the Nanobrick of Cement. Chem. Mater..

[44] Murgier S., Zanni H., Gouvenot D. (2004). Blast furnace slag cement: A 29Si and 27Al NMR study. C. R. Chim..

[45] Zhu Z., Wang Z., Zhou Y., Chen Y., Wu K. (2022). Nanoscale determination of calcium silicate hydrate (C-S-H) precursors crystallized at extreme early stage. Measurement.

[46] Xu Z., Gao J., Zhao Y., Li S., Guo Z., Luo X., Chen G. (2022). Promoting utilization rate of ground granulated blast furnace slag (GGBS): Incorporation of nanosilica to improve the properties of blended cement containing high volume GGBS. J. Clean. Prod..

